# Effect of Arsenic Exposure during Pregnancy on Infant Development at 7 Months in Rural Matlab, Bangladesh

**DOI:** 10.1289/ehp.11670

**Published:** 2008-10-24

**Authors:** Fahmida Tofail, Marie Vahter, Jena D. Hamadani, Barbro Nermell, Syed N. Huda, Mohammad Yunus, Mahfuzar Rahman, Sally M. Grantham-McGregor

**Affiliations:** 1 Child Development Unit, Clinical Sciences Division and Public Health Sciences Division, International Center for Diarrhoeal Disease Research, Dhaka, Bangladesh; 2 Institute of Environmental Medicine, Karolinska Institutet, Stockholm, Sweden; 3 Institute of Nutrition and Food Science, University of Dhaka, Dhaka, Bangladesh; 4 Department of Epidemiology, Mailman School of Public Health, Columbia University, New York, USA; 5 Centre for International Health and Development Institute of Child Health, University College London, London, United Kingdom

**Keywords:** Bangladesh cognitive function, infants, motor development, problem-solving tests, urinary arsenic

## Abstract

**Background:**

Exposure to arsenic-contaminated drinking water during pregnancy is associated with low birth weight and fetal loss, and there is concern that the infants’ development may be affected.

**Objective:**

We assessed the effects of *in utero* arsenic exposure during pregnancy on infants’ problem-solving ability and motor development.

**Methods:**

We conducted a large population-based study of nutritional supplementation with 4,436 pregnant women in Matlab, Bangladesh, an area of high-arsenic–contaminated tube wells. We measured arsenic concentration in spot urine specimens at 8 and 30 weeks of pregnancy. We assessed a subsample of 1,799 infants, born to these mothers, at 7 months of age on two problem-solving tests (PSTs), the motor scale of the Bayley Scales of Infant Development–II, and behavior ratings.

**Result:**

Arsenic concentrations in maternal urine were high, with a median (interquartile range) of 81 μg/L (37–207 μg/L) at 8 weeks of gestation and of 84 μg/L (42–230 μg/L) at 30 weeks. Arsenic exposure was related to many poor socioeconomic conditions that also correlated with child development measures. Multiple regressions of children’s motor and PST scores and behavior ratings, controlling for socioeconomic background variables, age, and sex, showed no significant effect of urinary arsenic concentration on any developmental outcome.

**Conclusion:**

We detected no significant effect of arsenic exposure during pregnancy on infant development. However, it is possible that other effects are as yet unmeasured or that effects will become apparent at a later age.

Arsenic is a widely distributed environmental pollutant with known carcinogenic and neurotoxicant effects [World Health Organization (WHO) 2001]. More than 100 million people worldwide have been estimated to be chronically exposed to drinking water containing high arsenic levels (Alaerts et al. 2001; National Research Council 2001). In Bangladesh, a nationwide survey initiated in 1998 indicated that about 35 million people were exposed to > 50 μg/L arsenic, which is the drinking water standard in Bangladesh, whereas 57 million had water exceeding the WHO guideline value of 10 μg/L (British Geological Survey 2001). Studies in school-age children have reported that arsenic exposure due to living near smelters in Mexico (Calderón et al. 2001; Rosado et al. 2007) and the United Sates (Wright et al. 2006) or drinking contaminated water in Taiwan (Tsai et al. 2003) and India (von Ehrenstein et al. 2007) is associated with deficits in children’s cognitive function. Two recent studies in Bangladesh in children 6 and 10 years of age (Wasserman et al. 2004, 2007) showed that arsenic concentration in their drinking water was related to deficits in global and performance IQ that were larger at 10 than at 6 years of age.

We are unaware of any prospective study examining the effect of exposure in pregnancy on offspring development. During pregnancy, transplacental transfer of arsenic occurs both in animals (Golub et al. 1998) and in humans (Concha et al. 1998). In animals, a high dose of arsenic is associated with detrimental effects on the developing embryo (Golub et al. 1998; National Research Council 1999; Wlodarczyk et al. 1996). In humans, exposure to high arsenic levels in drinking water is associated with reduction in birth weight (Hopenhayn et al. 2003; Huyck et al. 2007) and increase in fetal loss (Ahmad et al. 2001; Rahman et al. 2007). Considering the above, we hypothesized that arsenic exposure during pregnancy would be neurotoxic to the developing brain and would lead to behavioral changes in the offspring.

We conducted a large community-based randomized trial of the effects of food and micronutrient supplementation in pregnant women [Maternal and Infant Nutritional Intervention at Matlab (MINIMat) study] on birth outcomes and the development of their children in Matlab, a rural area of Bangladesh. We previously reported that in infants of undernourished mothers, food and micronutrient supplements added small benefits to their development at 7 months of age (Tofail et al. 2008). In the study area, 63% of all functioning tube wells were found to have > 50 μg/L arsenic in the water (Vahter et al. 2006). We obtained information on concurrent arsenic exposure of these women by measurements of arsenic in their urine collected twice during pregnancy (Vahter et al. 2006). The aim of this study was to examine the relationship between prenatal arsenic exposure and infants’ cognitive and motor development at 7 months of age.

## Materials and Methods

### Study area

The study area is in Matlab, a subdistrict of Chandpur, and is the field site of the International Centre for Diarrhoeal Disease Research, Bangladesh (ICDDR,B). It includes 142 villages covering a population of 220,000. It is located in the delta formed by the Meghna and the Ganges Rivers that lies in the east-central plain of Bangladesh, 53 km southeast of Dhaka. It is a poor rural area, and the main economic activities are farming, fishing, trading, and crafts. In this area, 95% of the population use tube wells for drinking water, and > 60% of the wells have arsenic levels > 50 μg/L (Rahman et al. 2006). ICDDR,B has maintained a Health and Demographic Surveillance System (HDSS) in the area to collect vital demographic and health information since 1966.

### MINIMat study

We identified pregnant women for the MINIMat study using the HDSS, in which all homes in the area were visited monthly and women were asked about their menstruation. If they reported amenorrhea at the time of a home visit and pregnancy was confirmed by urine tests and ultrasonogram, they were enrolled (*n* = 4,436). The women were randomized to two different prenatal interventions: They were first randomized to food consisting of a mixture of rice, lentils, molasses, and oil (two groups: early start or usual start) and then to three groups of micronutrient supplements from week 14 of gestation until delivery. The micronutrient groups received 60 mg iron + 400 μg folate, 30 mg iron + 400 μg folate, or 15 different vitamins and minerals containing iron and folate as well as iodine, zinc, selenium, copper, vitamins A, B_1_, B_2_, B_3_, B_6_, B_12_, C, D, and E. Further details of the dose have been published elsewhere (Tofail et al. 2008). Anthropometric measures of the women were taken on enrollment, and the infants’ weight, length, and head circumference were measured within 72 hr of birth.

### Study subjects

There were 3,267 live births from the MINIMat study, and we selected a subsample (*n* = 2,853) comprising all pregnant women who delivered live-born singleton infants between May 2002 and December 2003 for developmental assessments at the age of 7 months. This provided sufficient power to allow examination of interactions among treatments with at least 1 complete year of births to cover seasonal variation. We assessed 74% (2,116) of children. Of the 737 children not tested, 298 (40%) were away from home at the time, 197 (27%) refused to be tested (probably because of fear of venipuncture), 121 (16%) had died, 59 (8%) had moved residence, and 62 (9%) were sick at the time of testing. Of the 2,116 tested infants, 1,799 had information on their mothers’ urinary arsenic in pregnancy and complete socioeconomic data collected in the MINIMat study and HDSS.

### Measurements

#### Biochemical measures

We based exposure assessment on arsenic in urine, which reflects total exposure to inorganic arsenic from all sources. Spot urine samples were collected from the mothers at home at the time of pregnancy testing (gestational week 8, on average) and again at the clinic during the 30th gestational week. Urine was collected in cups and transferred to 24-mL acid-washed polyethylene containers. The urine samples were kept cold and transported to the hospital and deep-frozen to −70°C at the end of the day at the latest. They were subsequently transported (deep-frozen) to the Karolinska Institutet in Sweden for analysis of the total concentration of metabolites of inorganic arsenic, using the hydride generation atomic absorption spectroscopy method (Vahter et al. 2006). We adjusted the arsenic concentrations for variation in urine dilution by specific gravity, which we have found is less dependent on nutritional status and arsenic exposure than is creatinine adjustment (Nermell et al. 2008).

#### Socioeconomic status

On enrollment, information about family structure, parental characteristics, and socioeconomic conditions was collected by interview at home. The information included number of family assets (number owned from a list of possessions, e.g., television, radio, domestic animals, chairs, tables, beds, bicycle, rickshaw), deficits between income and expenditure (occasional or constant deficit in the previous month, yes/ no), number of children, and housing quality (floor, walls and/or roof made of mud, yes/no).

#### Anthropometry

On enrollment, mothers’ weights were measured with SECA electronic scales (Seca GmbH, Hamburg, Germany), which are accurate to 100 g, and their heights were measured to the nearest 0.1 cm using a locally manufactured height stick. We calculated mothers’ body mass index (BMI; weight in kilograms/height in square meters). Infants’ birth weight was measured using beam balance scales that were precise to 10 g, and their lengths were measured with locally made length boards. Head circumference was measured to the nearest 1 mm using nonstretchable tape.

At 7 months, the children were weighed and their lengths were measured at home. Research assistants were trained to carry out the anthropometric measurements according to standard procedures (WHO 1983). We converted the children’s heights and weights to standard scores using the WHO reference (WHO 2006).

#### Cognitive and motor function

The Bayley Scales of Infant Development–II (BSID-II) (Bayley 1993) has two scales: the Psychomotor Development Index (PDI) and the Mental Development Index (MDI). It is the most commonly used infant test and has been used previously in Bangladesh (Black et al. 2004; Hamadani et al. 2002; Tofail et al. 2006) and showed good interobserver and test–retest reliabilities. We assessed the children’s motor development with the motor scale. In the first 2 years of age, the motor scale tends to be more sensitive to biologic differences such as the effects of iron supplementation in iron-deficient infants (e.g., Lind et al. 2004; Moffatt et al. 1994) and protein energy supplementation in undernourished populations (Pollitt and Oh 1994) than is the mental scale. In the present study, it was sensitive to differences between children whose mothers received either multiple micronutrient supplementation or iron and folate only in pregnancy (Tofail et al. 2008). It was also related to the child’s gestational age and current nutritional status and mothers’ nutritional status.

We used two one-step means–end problem-solving tests (PSTs), Support and Cover, to assess the infants’ cognitive development. The original procedures of the PSTs were described by Piaget (1955), but the conduct and scoring of the tests were designed by Willatts (1984, 1999). In these tests, infants manipulate an intermediary to retrieve a goal (a toy). The Support test involves placing a long cloth on a table in front of the child and then placing a toy out of the child’s reach at the farthest end of the cloth. The infant has to pull the cloth to retrieve the toy. In the Cover test, a toy is covered with a cloth while the infant is watching. The infant is then required to remove the cloth to retrieve the toy. We videotaped these procedures and scored them later. We gave four trials in both PSTs. We scored three behaviors in each trial: cloth behavior (the way the child handled the cloth), fixation behavior (the way the child fixed his or her vision on the toy), and toy behavior (the way the child grasped the toy). We scored each behavior on a three-point scale: 0 for no evidence of intention, 1 for possible/ambiguous intention, and 2 for clear evidence of intention. We summed the scores for each behavior over the four trials to give a total score ranging from 0 to 24.

We chose the PSTs rather than the Bayley MDI, which is a global measure of development, because at this age the MDI does not correlate well with intelligence test scores in later childhood (Colombo 1993; Slater 1995). The PSTs measure specific cognitive functions, and early problem solving is related to IQ in later childhood (Slater 1995; Willatts 1997). The PSTs are sensitive to biologic and psychosocial differences in the first year of life, including birth weight in babies born at term (Meeks Gardner et al. 2003), supplementation with long-chain polyunsaturated fatty acids (Willatts et al. 1998a, 1998b), and the effects of 2 months of psychosocial intervention (Meeks Gardner et al. 2003). In the present sample, the test scores were sensitive to differences between infants whose mothers were supplemented with food early or later in pregnancy, and we achieved good test–retest and interobserver reliabilities (Tofail et al. 2008). The tests are relatively easy to perform and can be readily scored from videotape, which facilitates ongoing quality control in a large study. Therefore, if arsenic exposure *in utero* affects problem solving in infancy, we considered it likely that the effects would be detected.

#### Behavior

We also used a modified version of Wolke’s behavior ratings (Wolke et al. 1990) to assess infant’s behavior during the assessments. This instrument has five ratings with nine-point scales: activity level (very still = 1 to overactive = 9), emotional tone (unhappy = 1 to radiates happiness = 9), response to examiner in first 5 min (avoiding = 1 to friendly and inviting = 9), cooperation with the test procedure (resists all suggestions = 1 to always complies = 9), and the amount of vocalization (very quiet = 1 to constant vocalization = 9) of infants. These ratings have also been used before in Bangladesh (Hamadani et al. 2002; Tofail et al. 2006).

At the beginning of the study, we assessed interobserver reliabilities among the five testers with 20 infants. Intraclass correlations for scores of both Support and Cover PSTs and Bayley PDI were > 0.95 and for all behavior ratings were > 0.89. We assessed test–retest reliabilities before the start of the study. Test–retest intraclass correlations over 24 hr for both Support and Cover PST scores on 15 infants were > 0.70 and for Bayley PDI on 10 infants over a 7-day interval was 0.9. To maintain quality of testing, we assessed ongoing interobserver reliability weekly on 7% of all tests (PSTs and Bayley PDI were > 0.90 and for all behavior ratings were > 0.85).

### Procedure

Mothers were requested to bring their child to a subcenter for assessments of their child’s development at 7 months of age. All mothers were reimbursed for their travel costs and time, provided with snacks, and the infants were given a toy after the test session. Psychologists who were unaware of the children’s level of arsenic exposure assessed the children’s development in the presence of their mothers at one of four local clinics. We trained five psychologists to do the developmental assessments, and four of them rotated through each of the subcenters, spending 3 months in a center and then moving to the next one.

### Statistical analysis

We analyzed data using SPSS for Windows (version 10; SPSS Inc., Chicago, IL, USA). The distribution of urinary arsenic was skewed, so the data were log transformed. Urinary arsenic concentrations at 8 and 30 weeks’ gestation were moderately well correlated (*r* = 0.60, *n* = 1,799, *p* < 0.001), and we averaged the two values to give exposure throughout pregnancy. First, we categorized the urinary arsenic concentration into quartiles and conducted one-way analysis of variance (ANOVA) controlling for child’s age to examine the difference in developmental scores and behavior ratings by arsenic quartile. We then looked for potential confounders by correlating the sociodemographic variables with the developmental outcomes and with urinary arsenic concentration. We found no significant relationship between the nutritional supplementation group and urinary arsenic concentration (ANOVAs), and the supplement group did not modify the effect of arsenic on child development, so we did not further consider supplementation in any analyses. To examine the effect of arsenic exposure on infant development, we conducted multiple linear regression analyses of each developmental outcome, controlling for age and offering all covariates that were related to any of the child development measures in univariate analyses, and then entering mean urinary arsenic. We repeated all the regression analyses with the 8- and 30-week urinary arsenic concentrations.

### Ethics

All mothers were informed about the study and gave consent to participate. In a parallel study in the same area beginning February 2002, we screened all the tube wells for arsenic content (Rahman et al. 2006); we painted red those with water containing > 50 μg/L arsenic and advised people to collect water from green-painted wells with low arsenic concentrations. Unfortunately, the exposure in the study area remained high for most of the studied women (Vahter et al. 2006), generally because of the difficulties involved in mitigation. The ICDDR,B research and ethical review committees approved the study.

## Results

Maternal urinary arsenic in early and late pregnancy and developmental data were available for 1,799 infants. Birth size data were missing for 69 of these infants, mainly because their mothers were away for delivery. Anthropometric data at 7 months of age were missing from 255 children (17.6%) mainly because they were not at home at the time of the home visit.

### Sample characteristics

Table 1 shows the enrollment characteristics of the parents and characteristics of infants at birth and 7 months. The median (interquartile range) concentration of urinary arsenic at 8 weeks gestation was 81 (37–207) μg/L and at 30 weeks was 84 (42–230) μg/L.

Scores for Support differed by sex, with boys doing better than girls (11.38 ± 7 and 10.62 ± 7 respectively, *p* = 0.023). All three developmental outcomes were related to age at the time of testing (age and Support, *r* = 0.07; age and Cover, *r* = 0.08; age and PDI, *r* = −0.20; *p* < 0.01 for all). Among five behavior ratings, only cooperation was related to age of testing (age and cooperation, *r* = −0.05; *p* < 0.01). We therefore controlled for age when using these variables and sex when analyzing Support.

### Relation between arsenic and child development

Table 2 shows the mean developmental scores by arsenic quartile. None of the scores varied significantly by urinary arsenic quartile (analyses of covariance, group effect not significant), but the support test showed a significant linear trend (*p* < 0.05). Similarly, we found no difference in any of the behavior ratings by arsenic quartile (data not shown). Many socioeconomic variables and child characteristics were significantly correlated with both urinary arsenic and one or more child development measures (Table 3). We controlled for all variables significantly related to any child development variable in multiple regression analyses of each developmental outcome. Current length for age and weight for age were highly related to each other, so we chose length for age because it had slightly higher correlations with the development measures. For similar reasons, we offered birth length and head circumference and not birth weight. In all regression analyses, we entered age (and sex for support) in the first step. We entered fathers’ education; mothers’ education, BMI, and parity; housing; assets; income deficits; and child’s gestational age, birth length, head circumference, and current length *Z*-score in the second step, and then entered the urinary arsenic concentration in the third step (Table 4). Urinary arsenic was not significant in any of the regressions. Assets, fathers’ educational level, mothers’ BMI, and child’s gestational age, birth length, current height, sex, and age were significant covariates in one or more of the regressions. We examined possible interactions of arsenic with these covariates and sex. None were significant. We conducted analogous multiple regression analyses with the five behavior ratings and found no effect of urinary arsenic on any of them.

### Birth characteristics and current anthropometry

We repeated the regressions omitting measures of birth size and gestational age because it is possible that arsenic exposure affects child development through its effect on birth size and gestation. We also omitted current height for age because not only could arsenic exposure affect growth, but also 234 children had missing anthropometric data. The effect of arsenic exposure remained nonsignificant.

## Discussion

After controlling for socioeconomic differences, we failed to detect any deficit in problem-solving abilities, motor development, or behavior in tests of 7-month-old infants who were prenatally exposed to a wide range of arsenic concentrations. This was a large population-based study, involving 1,799 infants, and we had individual measures of maternal urinary arsenic concentrations at two periods in pregnancy. In addition, we measured a large number of variables that could affect child development and took them into account in the analyses. The developmental tests had good test–retest and interobserver reliabilities and appeared to be sensitive. In the multiple regressions, household assets, fathers’ education, maternal nutritional status, and child’s birth size, gestational age, current nutritional status, sex, and age were significantly independently related to one or more of the child development outcomes. It is therefore reasonable to conclude that the lack of any marked effect of arsenic exposure in pregnancy on infant motor development and problem solving is probably a valid finding.

The lack of detectable effect is encouraging considering the high levels of arsenic exposure. However, we need to be cautious when interpreting the findings because other unmeasured cognitive functions might have been affected. Furthermore, we examined these children at only one age, and it remains possible that effects could appear at a later age when new functions develop. We were unable to find another prospective study that examined the effect of intrauterine exposure of arsenic on infant development.

A recent study took a retrospective history of prenatal exposure to arsenic-contaminated drinking water and found no effect on school-age children’s cognition (von Ehrenstein et al. 2007). However, cognitive deficits in schoolchildren currently exposed to arsenic-contaminated drinking water have been found in different countries (Rosado et al. 2007; Wang et al. 2007; Wasserman et al. 2004), although the specific cognitive functions affected have varied and the effect size was generally small. It may be that duration of exposure is important. Previous studies in Bangladesh found a smaller effect of arsenic exposure in 6-year-olds (Wasserman et al. 2007) than in 10-year-olds (Wasserman et al. 2004). In the present study, arsenic concentration in urine was related to a wide range of socioeconomic variables. The association between arsenic exposure and circumstances of poverty is a common finding (Rahman et al. 2006), and the effect of poverty on child development is well documented and generally increases with age (Grantham-McGregor et al. 2007). It is difficult to measure all factors associated with poverty that affect child development. For example, few studies of arsenic exposure in school-age children have measured stimulation in the home, which is an important determinant of child development. However, the previous Bangladeshi study of 6-year-olds attempted to take stimulation into account and still found a small deficit in cognitive function associated with arsenic exposure (Wasserman et al. 2007).

Arsenic exerts its toxic effects at least partly via oxidative effects (Chattopadhyay et al. 2002). In the present study, all mothers received folate in pregnancy, and a possible explanation for the lack of a detectable effect is that there was some protection due to folate, which has antioxidant properties (Mukherjee et al. 2006). Another protective mechanism is the induced metabolism of arsenic in pregnancy (Vahter 2007). Inorganic arsenic, the main form in the drinking water, is methylated via one-carbon metabolism, producing methylarsonic acid (MMA) and dimethylarsinic acid (DMA), which are the main metabolites excreted in urine (Vahter 2002). Although a high percentage of MMA in urine is considered a risk factor for a wide range of toxic effects of arsenic (e.g., Li et al. 2008; Tseng 2007), a high percentage of DMA is associated with increased rate of excretion and fewer health risks. We found that the mothers of the studied infants had remarkably efficient methylation of arsenic already in early pregnancy (Li et al. 2008), and it is likely that further improvement occurred with advancing gestation (Concha et al. 1998; Hopenhayn et al. 2003).

The present findings are likely to reflect mainly intrauterine exposure, because arsenic readily crosses the placenta and breast milk excretes very little arsenic (Concha et al. 1998). In a subsample in this cohort, the median concentration of arsenic in breast milk was 1 μg/L (Fangstrom et al. 2008). In this cohort, exclusive breast-feeding lasted 4 months on average, and essentially all children were partly or predominantly breast-fed at the time of the testing at 7 months (Saha et al. 2008). However, around half the children were taking semisolids or solids at 6 months of age, mostly begun after 5 months. Therefore, a limitation to the study is that the children would have had some small exposure to other sources of arsenic.

## Conclusion

In conclusion, we failed to detect significant effects of prenatal arsenic exposure on infants’ problem-solving abilities, motor development, and behavior. It is possible that other cognitive functions are affected and that effects may appear at a later age. We intend to follow these children to determine if deficits occur in the future. There remains an urgency to reduce arsenic exposure in pregnancy owing to the increase in prenatal and infant mortality previously reported in this area (Rahman et al. 2007).

## Figures and Tables

**Table 1:** Characteristics of families on enrollment and infants at birth and 7 months (*n* = 1,799).

Variable	No.	Value
Family characteristics		
Poor housing	1,799	21
Occasional or constant income-expenditure deficit	1,799	18.7
Assets (median)	1,799	−0.05 ± 2.3 (0.32)
Fathers’ education (% < 5th grade)	1,784	42
Mothers’ education (% < 5th grade)	1,799	44.5
Mothers’ age (years)	1,799	26.4 ± 6.0
Mothers’ parity (% primipara)	1,796	32
Mothers’ BMI (kg/m^2^ )	1,789	20.2 ± 2.7
Gestational age (weeks)	1,799	39.2 ± 1.6
Median of mean arsenic concentration in mothers’ urine (interquartile range) (μg/L)		
Gestational week 8 and 30	1,799	98 (45–218)
Gestational week 8	1,799	81 (37–207)
Gestational week 30	1,799	84 (42–230)
Infant’s birth anthropometry		
Weight (g)	1,728	2,696 ± 393
Length (cm)	1,728	47.8 ± 2.1
Head circumference (cm)	1,728	32.5 ± 1.7
Infant’s characteristics at 7 month testing		
Age (months)	1,799	7.4 ± 0.3
Height for age (*Z*-score)	1,542	−1.4 ± 1.05
Weight for age (*Z*-score)	1,542	−1.2 ± 1.1
Weight for height (*Z*-score)	1,542	−0.3 ± 1.1

Values are mean ± SD or percent.

**Table 2:** Raw scores (mean ± SD) of infant PST (Cover and Support) and PDI by quartiles of maternal mean urinary arsenic concentration^a^ during pregnancy.

Arsenic exposure (μg/L)	No.	Total Cover	Total Support	PDI
0–45.0	450	12.9 ± 7.0	11.5 ± 7.6	103.2 ± 15.5
45.1–97.7	450	12.7 ± 7.1	11.4 ± 7.9	102.8 ± 16.2
97.8–218	449	13.4 ± 6.8	10.9 ± 7.3	103.3 ± 15.0
> 218	450	12.6 ± 7.2	10.4 ± 7.8	103.4 ± 15.4
Total	1,799	12.9 ± 7.0	11.0 ± 7.6	103.2 ± 15.5
Group difference^b^				
*p*-Value	0.3	0.1	0.9	
Linear trend significance	0.8	0.018	0.6	

a Mean of concentrations at gestational weeks 9 and 30.

b ANOVA controlling for age (Cover and PDI) and age and sex (Support).

**Table 3:** Correlations among maternal urinary arsenic concentration, infant developmental outcomes, socioeconomic variables, and child characteristics.^a^

	Mothers’ urinary arsenic^b^			Developmental outcome		
Variable	8 weeks (log)	30 weeks (log)	Mean of 8 and 30 weeks (log)	Cover^c^	Support^d^	PDI^c^
Mothers’ BMI on enrollment (log)	−0.08** (*n* = 1,791)	−0.09** (*n* = 1,791)	−0.09** (*n* = 1,791)	0.06* (*n* = 1,787)	0.06* (*n* = 1,787)	0.08** (*n* = 1,787)
Parity	0.04 (*n* = 1,797)	−0.01 (*n* = 1,797)	−0.02 (*n* = 1,797)	−0.04 (*n* = 1,793)	−0.05* (*n* = 1,793)	0.06* (*n* = 1,793)
Mother’s education (years in school)	−0.12** (*n* = 1,799)	−0.12** (*n* = 1,799)	−0.13** (*n* = 1,799)	0.08** (*n* = 1,795)	0.06** (*n* = 1,795)	0.03 (*n* = 1,795)
Father’s education (years in school)	−0.12** (*n* = 1,786)	−0.10** (*n* = 1,786)	−0.12** (*n* = 1,786)	0.12** (*n* = 1,782)	0.08* (*n* = 1,782)	0.05* (*n* = 1,782)
Income/expenditure deficit	−0.02 (*n* = 1,799)	0.01 (*n* = 1,799)	−0.01 (*n* = 1,799)	0.07* (*n* = 1,795)	0.06* (*n* = 1,795)	0.04 (*n* = 1,795)
Assets	−0.14** (*n* = 1,799)	−0.08** (*n* = 1,799)	−0.12** (*n* = 1,799)	0.08* (*n* = 1,795)	0.07* (*n* = 1,795)	0.02 (*n* = 1,793)
Housing	−0.20** (*n* = 1,799)	−0.17** (*n* = 1,799)	−0.21** (*n* = 1,799)	0.07** (*n* = 1,795)	0.05* (*n* = 1,795)	0.03 (*n* = 1,795)
Birth weight (g)	−0.02 (*n* = 1,730)	−0.04 (*n* = 1,730)	−0.04 (*n* = 1,730)	0.13** (*n* = 1,726)	0.11** (*n* = 1,726)	0.22** (*n* = 1,726)
Length at birth (cm)	−0.02 (*n* = 1,728)	−0.05* (*n* = 1,728)	−0.04 (*n* = 1,728)	0.15** (*n* = 1,724)	0.11** (*n* = 1,724)	0.17** (*n* = 1,724)
Gestational age (weeks)	−0.02 (*n* = 1,798)	−0.06* (*n* = 1,798)	−0.05* (*n* = 1,798)	0.17** (*n* = 1,794)	0.17** (*n* = 1,794)	0.26** (*n* = 1,794)
Head circumference at birth (cm)	−0.05* (*n* = 1,736)	−0.05* (*n* = 1,736)	0.07* (*n* = 1,736)	0.09** (*n* = 1,732)	0.10** (*n* = 1,731)	0.13** (*n* = 1,732)
Length for age (*Z-*score at 7 months)^d^	−0.03 (*n* = 1,542)	−0.06* (*n* = 1,542)	−0.05* (*n* = 1,542)	0.13** (*n* = 1,540)	0.12** (*n* = 1,539)	0.16** (*n* = 1,540)
Weight for age (*Z*-score at 7 months)^d^	−0.04 (*n* = 1,542)	−0.06* (*n* = 1,542)	−0.05 (*n* = 1,542)	0.12** (*n* = 1,538)	0.11** (*n* = 1,537)	0.15** (*n* = 1,538)
Weight for length (*Z*-score at 7 months)^d^	−0.03 (*n* = 1,542)	−0.03 (*n* = 1,542)	−0.02 (*n* = 1,542)	0.04* (*n* = 1,538)	0.03* (*n* = 1,537)	0.04* (*n* = 1,538)

a *n* = 1,799–1,724, depending on available data (see Table 1), except *n* = 1,542–1,537 for anthropometry at 7 months.

b Pearson’s correlations.

c Controlling for age.

d Controlling for age and sex.

* *p* < 0.05;

** *p* < 0.001.

**Table 4:** Significant regression coefficients (B), standard error (SE), and 95% confidence interval (CI) from multiple regressions of Support and Cover PSTs (*n* = 1,467) and PDI (*n* = 1,465).

	B ± SE (95% CI)		
Variable	Cover	Support	PDI
Age (months)	0.03 ± 0.03 (−0.02 to 0.09)	0.05 ± 0.03 (−0.01 to 0.1)	−0.5 ± 0.05 (−0.6 to −0.4)**
Sex	—	−1.1 ± 0.4 (−1.6 to −0.2)*	—
Assets	—	0.2 ± 0.09 (−1.6 to −0.2)*	—
Father’s education	0.2 ± 0.04 (0.2–0.3)**	0.1 ± 0.04 (0.03–0.2)**	—
Gestational age (weeks)	0.5 ± 0.1 (0.2–0.7)**	0.6 ± 0.1 (0.2–0.7)**	2.2 ± 0.5 (1.7–2.7)**
Length at birth (cm)	0.3 ± 0.1 (0.2–0.7)**	—	—
Length at 7 months (*Z*-scores)	—	0.4 ± 0.2 (0.05–0.8)*	1.3 ± 0.4 (0.6–2.1)*
Mothers’ BMI (kg/m^2^ )	—	0.1 ± 0.1 (0.005–0.3)*	0.4 ± 0.1 (0.1–0.7)*
Mothers’ urinary arsenic (mean of weeks 8 and 30, μg/L)	0.4 ± 0.4 (−0.4 to 1.3)	−0.6 ± 0.5 (−1.5 to 0.4)	0.9 ± 0.9 (−0.9 to 2.7)
*R*^2^	0.05	0.04	0.12

The model was as follows: step 1, age (and sex for support) entered; step 2, mothers’ and fathers’ education (years completed), housing, assets, income, mothers’ BMI and parity, and child’s birth length and head circumference, gestational age, and length in *Z*-scores at 7 months offered; step 3, mean of mothers’ urinary arsenic (log) entered. The PSTs are raw scores. The regression coefficients (B) of the PSTs indicate the amount of change in raw scores per unit of the independent variable (one standard score for Cover is 7 and for Support is 7.6).

* *p* < 0.05;

** *p* < 0.001.
